# A Proposition

**Published:** 1873-08

**Authors:** 


*A Proposition.*

Dr. W. T. Taylor, of Kentucky, writes: "I would that I had influence to induce every physician in the whole South to subscribe for the RECORD. While fully up to the standard of the best medical journals, and surpassing the most of them, still, the larger the patronage, the more ability will the editors have of further improving and enlarging it.

"I will be one of two hundred who will send five new subscribers, each accompanied by the cash, between this and Christmas. I know there are many more than the above number who can easily get that many names in the time. Besides this, I do not believe there is a single subscriber to the RECORD who cannot, if they would, get at least *one* new name, and that, too, without difficulty. This would give at least one thousand new names with which to commence the new year. Shall it be done?"

The above proposition we place before our readers. Are they prepared to go to work on Dr. Taylor's proprosition? Out of our large list, surely there are two hundred *warm, working* friends of the RECORD who will try to secure five subscribers each before Christmas next. We will, however, make this proposition: If two hundred of our friends will agree to pledge themselves to send us five new subscribers by the first of January, we, on our part, will pledge ourselves to enlarge the size of the journal without additional cost, and will give a premium (in books, instruments, or anything else desired), to the value of SEVENTY-FIVE DOLLARS to the person getting the largest number of names over five; FIFTY DOLLARS to the next, and TWENTY-FIVE DOLLARS to the next. Go to work, friends; you will have until January to get up your list of names.

ATTENTION is called to the advertisements of two first-class medical colleges, which appear in this issue. In our next, we will have several others—colleges having the "men and the means" for imparting thorough medical education.

